# Invasive Ventilation Strategies in Neonates

**DOI:** 10.1007/s13312-025-00094-6

**Published:** 2025-06-03

**Authors:** Jogender Kumar, Praveen Kumar, Vineet Bhandari

**Affiliations:** 1 Department of Pediatrics, Post Graduate Institute of Medical Education and Research, Neonatal Unit, Chandigarh, 160012 India; 2 Division of Neonatology, Department of Pediatrics, Cooper Medical School of Rowan University, The Children’s Regional Hospital at Cooper, Camden, NJ 08103 USA

**Keywords:** High-frequency ventilation, Pressure-targeted ventilation, Preterm infant, Synchronized intermittent mandatory ventilation, Volume-targeted ventilation

## Abstract

We provide evidence-based recommendations and clinical guidance on strategies for invasive mechanical ventilation in neonates until successful extubation in the neonatal intensive care unit. A systematic search of the PubMed, Embase, and CENTRAL databases was performed to identify relevant published literature from the past five years. A critical review of the current literature was conducted to provide context-specific recommendations. We discuss the various modes of invasive mechanical ventilation in neonates, with specific recommendations for neonates with persistent pulmonary hypertension, congenital heart disease, congenital diaphragmatic hernia, pneumonia, meconium aspiration syndrome, air leak syndromes, evolving and established bronchopulmonary dysplasia, apnea, and very preterm infants with respiratory distress. Practical guidance for the initiation, titration, and weaning of volume-targeted ventilation is also provided. Synchronized patient-triggered modes (synchronized intermittent mandatory ventilation + pressure support ventilation/assist control ventilation) and volume-target/guarantee modes are the preferred modes of invasive mechanical ventilation in neonates with respiratory distress.

## Introduction

Respiratory distress is the most common indication for neonatal intensive care unit (NICU) admission and mechanical ventilation [1, 2]. At present, noninvasive ventilation (NIV) is considered the first-line approach for managing neonatal respiratory distress. A detailed description of NIV has been provided elsewhere. Although efforts have been made to avoid invasive mechanical ventilation as much as possible, nearly 40% of very low birth weight and 80% of extremely premature infants (< 28 weeks gestational age or GA) require invasive mechanical ventilation at some point in time [1, 3–5].

Invasive mechanical ventilation refers to the delivery of positive pressure to the lungs via an endotracheal or a tracheostomy tube. The primary goal of invasive mechanical ventilation is to correct compromised lung function, restore adequate gas exchange, and reduce the work of breathing. Invasive mechanical ventilation is a major cause of ventilator-induced lung injury (VILI). Therefore, one of the key goals of invasive mechanical ventilation is to reduce the duration of invasive mechanical ventilation by early extubation as soon as the infant is deemed ready. In neonates, the common indications for invasive mechanical ventilation are inadequate or absent respiratory efforts, recurrent apnea, failure or contraindication of NIV, severe lung disease, and postsurgical conditions. In this article, we provide an evidence-based practical approach for neonatal invasive mechanical ventilation.

## Evidence Acquisition

We searched PubMed, Embase, and CENTRAL for relevant original articles, systematic reviews, and evidence-based guidelines regarding various aspects of invasive ventilation in neonates. The search strategy included keywords related to the population (neonate, infant, preterm), intervention (invasive ventilation, volume-targeted/guaranteed ventilation, high-frequency ventilation or HFV, and pressure support ventilation), specific conditions (persistent pulmonary hypertension of the newborn or PPHN, congenital heart disease or CHD, congenital diaphragmatic hernia or CDH, pneumonia, meconium aspiration syndrome or MAS, air leak syndromes, those with apnea and very preterm with respiratory distress, and bronchopulmonary dysplasia or BPD), and outcomes (extubation, ventilation days). To remain current, the search was limited to the last five years (2019–2024) unless there was no update in the past five years. We also searched the guidelines issued by various societies [American Academy of Pediatrics (USA) or AAP, National Institute for Health and Care Excellence (UK), NICE, National Neonatology Forum (India), NNF, European Standards of Care for Newborn Health [ESCNH], etc.) on relevant topics. All relevant retrieved articles were collated, critically analyzed, appraised, and summarized according to the objective of the review. We first evaluated the most recent evidence-based guidelines from prominent societies and the most recent systematic reviews and meta-analyses of randomized clinical trials (RCTs) to provide up-to-date evidence. We searched for evidence pertaining to low- and middle-income countries (LMICs), such as India, to make it context-specific. We provide a summary of the most recent evidence in Table 1. In situations in which evidence does not exist, the authors provide expert opinions. Other crucial aspects of invasive mechanical ventilation, such as humidification, physiotherapy, sedation, infection control, and blood gas monitoring, are beyond the scope of this article and can be accessed elsewhere [6].

**Table 1:** Evidence summary for invasive ventilatory support in neonates

Authors/comparison	No. of participants (studies)	Outcomes	Effect estimate RR (95% CI)	Certainty of evidence	Remarks
*Comparison of various modes of invasive mechanical ventilation*					
Greenough, et al. (2016) Assist control/SIMV mode of ventilation versus non-synchronized IMV mode of ventilation [9]	1790 (6 RCTs)	BPD	1.17 (0.94–1.47)	Low	No effect on air leaks, IVH and extubation failure
	1463 (5 RCTs)	Duration of ventilation (hours)	MD: − 38.3(− 53.9 to − 22.7)	Low	
Greenough, et al. (2016) Assist control mode of ventilation versus SIMV mode [9]	120 (3 RCTs)	Duration of weaning (hours)	MD: − 42.4 (− 94.4 to 9.6)	Very low	No difference in air leaks and extubation failure
	2782 (4 RCTs)	Weaning failure	0.78 (0.31–1.93)	Very low	
Greenough, et al. (2016) PS + SIMV mode versus SIMV alone [9]	107 (1 RCT)	Death	0.41 (0.08–2.01)	Very low	No difference in BPD, air leaks, and IVH
Greenough, et al. (2016) PSV versus SIMV [9]	60 (1 RCT)	Extubation failure	1.09 (0.57–2.07)	Very low	No difference in BPD and air leaks
Batra, et al. (2023) Any Assisted Ventilation modes versus SIMV alone [10]	339 (7 RCTs)	Weaning duration (hours)	MD: − 22.7 (− 44.3 to–1.01)	Very low	No difference in mortality, BPD, Extubation failure, or any other outcome
Klingenberg, et al. (2017) Volume-targeted ventilation (VTV) versus pressure-limited ventilation (PLV) [11]	584 (8 RCTs)	Death or BPD	0.73 (0.59–0.89)	Moderate	Lesser incidence of hypocarbia. No difference in other outcomes
	620 (9 RCTs)	BPD	0.68 (0.53–0.87)	Low	
	736 (12 RCTs)	Duration of PPV (days)	MD: − 1.35 (− 1.83 to − 0.86)	Moderate	
	825 (13 RCTs)	Pneumothorax	0.52 (0.31–0.87)	Moderate	
	712 (10 RCTs)	Severe IVH	0.53 (0.37–0.77)	Moderate	
Cools, et al. (2015) Elective HFOV versus Conventional ventilation for acute RDS [17]	3329 (17 RCTs)	Death or BPD	0.90 (0.84–0.97)	Very low	No difference in mortality alone, air leaks, and other outcomes
	2781 (12 RCTs)	Retinopathy of Prematurity	0.81 (0.70–0.93)	Very low	
Bhuta, et al. (2000) Rescue HFOV versus Conventional ventilation [18]	170 (1 RCT)	New onset of any air leak	0.73 (0.55–0.96)	Very low	No difference in mortality, severe air leaks, and severe IVH
	170 (1 RCT)	Any grade IVH	1.77 (1.06–2.96)	Very low	
Rojas- Reyes, et al. (2015) Rescue HFJV versus conventional ventilation [19]	166 (1 RCT)	Mortality before cross-over	0.66 (0.45–0.97)	very low	No difference in overall mortality and other outcomes

BPD Bronchopulmonary dysplasia; CI Confidence interval; HFOV High-frequency oscillatory ventilation; HFJV High-frequency jet ventilation; IVH Intraventricular hemorrhage; MD Mean Difference; PPV Positive pressure ventilation; RCT Randomized controlled trial; PS Pressure support; PSV Pressure support ventilation; RDS Respiratory distress syndrome; RR Risk ratio; SIMV Synchronized intermittent mandatory ventilation

## Results

**Fig. 1:**
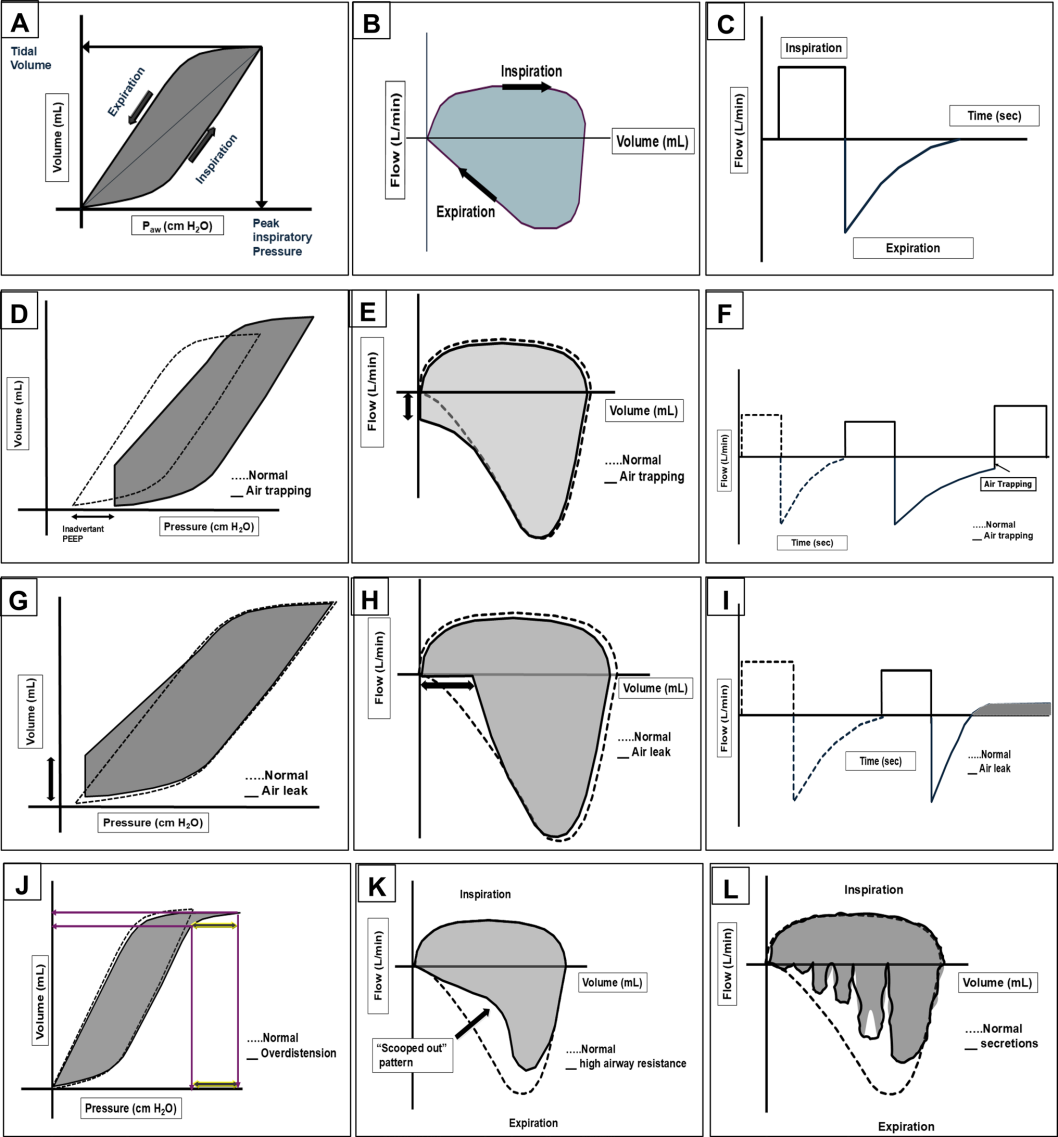
Pulmonary graphics showing normal pressure–volume and flow–volume loops (**A** and **B**), normal flow-time scalar (**C**), air trapping (**D**-**F**), air leak (**G**-**I**), overdistension (**J**), high airway resistance (**K**), and secretions (**L**)

In this section, we briefly describe the various modes of invasive mechanical ventilation along with their evidence (Table 1) and salient features of the different modes of invasive mechanical ventilation (Table 2). A pictorial representation of pulmonary graphics along with common problems is provided (Fig. 1) for easy understanding of the readers. General guidance for initiating and titrating volume-targeted ventilation is also provided (Box 1). In Table 3, we provide clinical guidance for ventilation strategies for patients with PPHN, CHD, CDH, pneumonia, MAS, air leak syndromes, apnea, and very preterm infants with respiratory distress as well as evolving and established BPD.

**Table 2:** Salient features of different modes of invasive ventilation

Mode	Trigger	Limit	Cycling	Synchronization	Parameters to set
Synchronized intermittent mandatory ventilation	Patient or ventilator	Pressure or volume	Flow (for spontaneous) or Time (for mandatory)	Synchronizes mandatory breaths with patient effort	Tidal volume (Vt) or pressure (PIP), rate, FiO₂, PEEP, trigger sensitivity
Intermittent mandatory ventilation	Patient or ventilator	Pressure or volume	Time (mandatory breaths), flow (spontaneous)	No synchronization, mandatory breaths delivered independently of patient effort	Tidal volume (Vt) or pressure (PIP), rate, FiO₂, PEEP
Pressure support ventilation	Patient (flow or pressure)	Pressure	Flow (when inspiratory flow drops to a preset level)	Patient-triggered breaths ensure synchronization	Pressure support, PEEP, FiO₂ trigger sensitivity, cycling off threshold
Assist control (A/C)	Patient or ventilator	Pressure or volume	Time (if time-triggered) or Flow (if patient-triggered)	Every patient-triggered breath receives full support (fully synchronized)	Tidal volume (Vt) or pressure (PIP), rate, FiO₂, PEEP trigger sensitivity

FiO_2_ Fraction of inspired oxygen; PIP Peak inspiratory pressure; PEEP Positive end-expiratory pressure

Explanations: **Trigger**: What initiates a breath (patient effort or time-based initiation by ventilator). **Limit**: What controls or caps the inspiratory phase (pressure or volume). **Cycling**: What ends the inspiratory phase (time, flow, or pressure). **Trigger Sensitivity**: Adjusts how easily the ventilator detects the patient’s effort

**Box 1:** General guide for volume-targeted ventilation

*Initiation*
Initiate volume-targeted ventilation (VTV) as the primary mode of invasive ventilation
Use synchronized modes (Assist control/SIMV + PS)
Select appropriate PEEP as per the underlying disease condition
Select appropriate target tidal volume (as per weight, underlying diagnosis)
Set PIP limit 3–5 cm H_2_O above expected PIP requirement
Check working of flow-sensor
Check for air leak and ET size
Check for work of breathing and SpO_2_. Adjust tidal volume (in steps of 0.5 mL/kg) if required
*Titration*
Use pulmonary graphics to titrate the PEEP and tidal volume
Monitor pH and try to keep it slightly acidotic
Adjust settings as per work of breathing, saturation and blood gases
*Weaning*
It is an auto weaning mode. PIP automatically comes down
Maintain pH between 7.25 and 7.35 to ensure triggering
Do not lower targeted tidal volume unless absolute necessary
See if the infant meets all criteria of the extubation (Box 2). If so, extubate

**Table 3:** Suggested settings for conventional modes of invasive ventilation for typical clinical scenarios^*a*^

Clinical scenario	Volume-target ventilation (VTV)^*b*^		SIMV settings	Remarks
	Initial target tidal volume	Initial PEEP and PIP Limits		
Preterm with RDS	4–5 mL/kg (Up to 6 mL/kg in < 750 g)	PEEP: 6–8 cm H_2_O PIP: 22–24 cm H_2_O	*Mild to moderate disease* PEEP: 5–6 cm H_2_O PIP: 16–20 cm H_2_O Ti: 0.35 s Rate: 40/minute *Severe disease* PEEP: 6–8 cm H_2_O PIP: 18–24 cm H_2_O Ti: 0.30 s Rate: 40–50/minute	In RDS, there is rapid change in compliance with surfactant administration; hence, PEEP needs to be adjusted rapidly No need to change the PIP limit and V_T_
Apnea/normal lung ventilation	4–5 mL/kg	PEEP: 4–5 cm H_2_O PIP: 16–18 cm H_2_O	PEEP: 4–5 cm H_2_O PIP: 12–14 cm H_2_O Ti: 0.35- 0.40 s Rate: 25–30/minute	
Evolving BPD	5–6 mL/kg	PEEP: 6–7 cm H_2_O PIP: 24–26 cm H_2_O	PEEP: 6–7 cm H_2_O PIP: 20–22 cm H_2_O Ti: 0.45- 0.5 s Rate: 30/minute	
Established/severe BPD	7–10 mL/kg	PEEP: 8–10 cm H_2_O PIP: 26–28 cm H_2_O	PEEP: 8–10 cm H_2_O PIP: 22–24 cm H_2_O Ti: 0.5–0.7 s Rate: 20–30/minute	Severe cases may require high PEEP (up to 15 cmH_2_O) and PIP (32–36 cmH_2_O)
MAS without ARDS	5–6 mL/kg	PEEP: 5–6 cm H_2_O PIP: 22–24 cm H_2_O	PEEP: 5–6 cm H_2_O PIP: 18–20 cm H_2_O Ti: 0.4–0.45 s Rate: 35–40/minute	
Pneumonia	5–6 mL/kg	PEEP: 5–7 cm H_2_O PIP: 18–24 cm H_2_O	*Mild to moderate disease* PEEP: 5–6 cm H_2_O PIP: 15–18 cm H_2_O Ti: 0.35–0.45 s Rate: 35–40/minute *Severe disease* PEEP: 6–8 cm H_2_O PIP: 18–24 cm H_2_O Ti: 0.30 s Rate: 40–50/minute	Severe pneumonia, if bilateral should be treated like ARDS
Air leak syndrome (PIE/PTX/PNM/Pneumopericardium)	4–5 mL/kg	PEEP: 3–5 cm H_2_O PIP:18–24 cm H_2_O	PEEP: 3-5 cm H_2_O PIP: 15–20 cm H_2_O Ti: 0.30–0.35 s for preterm and 0.30–0.40 for term infants Rate: 40–60/minute	Keep PIP < 25 cm H_2_O. Consider Early rescue HFV, if available
PPHN with Parenchymal disease	4–6 mL/kg	PEEP: 6–8 cm H_2_O PIP: 22–24 cm H_2_O	PEEP: 6–8 cm H_2_O PIP: 18–24 cm H_2_O Ti: as per disease pathology Rate: 40–50/minute	For PPHN with BPD, follow the suggested settings for BPD. Consider alternative therapies (iNO) as per Oxygenation Index values
PPHN with minimal/no parenchymal disease	4–5 mL/kg	PEEP: 4–5 cm H_2_O PIP: 16–18 cm H_2_O	PEEP: 4–5 cm H_2_O PIP: 12–14 cm H_2_O Ti: 0.35–0.45 s Rate: 40–45/minute	
Congenital cardiac disease (increased PBF)	5–6 mL/kg	PEEP: 5–8 cm H_2_O PIP: 20–24 cm H_2_O	PEEP: 5–8 cm H2O PIP: 16–20 cm H2O Ti: 0.35–0.45 s Rate: 35–40/minute	High PEEP is required in increased PBF. Titrate CO_2_ to control PBF
Congenital cardiac disease (reduced PBF)	4–5 mL/kg	PEEP: 4–5 cm H_2_O PIP: 16–18 cm H_2_O	PEEP: 4–5 cm H_2_O PIP: 12–14 cm H_2_O Ti: 0.35- 0.45 s Rate: 40–45/minute	
Lung Hypoplasia and CDH	4.5–6 mL/kg	PEEP: 3–5 cm H_2_O PIP: 18–24 cm H_2_O	PEEP: 3–5 cm H_2_O PIP: 18–24 cm H_2_O Ti: 0.25–0.40 s Rate: 40–60/minute	In CDH, the best TTV is not known. Until further evidence is available, PCV may be preferred as the initial mode. Consider rescue HFV, if available

ARDS Acute respiratory distress syndrome; BPD Bronchopulmonary dysplasia; CDH Congenital diaphragmatic hernia; HFOV High-frequency oscillatory ventilation; HFV High-frequency ventilation; iNO Inhaled nitric oxide; MAP Mean airway pressure; MAS meconium aspiration syndrome; PBF Pulmonary blood flow; PCV Pressure-controlled ventilation; PEEP Positive end-expiratory pressure; PIE Pulmonary interstitial emphysema; PIP Peak inspiratory pressure; PPHN Persistent pulmonary hypertension; PNM Pneumomediastinum; PTX Pneumothorax; RDS Respiratory distress syndrome; SIMV Synchronized intermittent mandatory ventilation; Ti Inspiratory time; TTV Targeted tidal volume; V_t_ Tidal volume; VTV Volume-targeted ventilation

^**a**^These are generic settings as a clinical guide; they should be individualized based on the clinical condition and postmenstrual age

^**b**^If changing from PCV to VTV, the target volume should be the same as generated by PCV. PIP limit should be 3–5 cm H_2_O above the PIP set in PCV and the PEEP should be same as PCV

### Modes of Invasive Mechanical Ventilation

#### Conventional Mechanical Ventilation (CMV)

The basic modes of mechanical ventilation depend on the manner in which the ventilator triggers the delivery of mechanical inflation, regulates (limits) inflation, and cycles it into expiration. If the trigger is the patient’s effort detected by the ventilator, it is called “assist” mode. If a triggering happens after a prefixed time limit, it is called “control” mode. Inflation, which is the volume delivered to the lung, is limited by the pressure limit set by the user and lung compliance in pressure-controlled ventilation or pressure-limited ventilation (PLV). It is limited by the volume limit set by the user for volume-controlled or volume-limited ventilation (VLV). In PLV mode, the primary control variable is inflation pressure, and the volume of gas entering the lungs (tidal volume, V_T_) is the dependent variable. In this mode, the pressure is fixed, and V_T_ fluctuates. In VLV, the gas delivery volume (V_T_) is the primary control variable, and the pressure is the dependent variable. In neonates, PLV is the usual mode used because of the use of uncuffed endotracheal tubes and a very small V_T_. To take advantage of the benefits of VLV and minimize the disadvantages of PLV, volume-targeted/guarantee ventilation (VTV, VG) is added to the PLV. Essentially, VTV is a pressure-controlled modality of ventilation with automatic adjustment of the peak inspiratory pressure (PIP) to target a user-set V_T_ measured at the airway opening [7, 8]. The ventilator can be made to cycle into expiration at a time set by the user (time-cycled), or at a preset drop in inspiratory flow set by the user (flow-cycled).

There are four basic modes of CMV: intermittent mandatory ventilation (IMV), synchronized intermittent mandatory ventilation (SIMV), assist control (AC), and pressure support ventilation (PSV). With the availability of synchronized modes, IMV is no longer used because it is known to cause patient discomfort and prolong ventilation [9]. The remaining three are synchronized modes that differ in terms of the trigger (time, flow), control (volume, pressure), and cycling (time, flow) (Table 2). Here, we briefly describe these three modes, along with the volume ventilation.

In SIMV, the ventilator delivers a set number of mechanical inflations at a prescribed V_T_ (or pressure) that are synchronized with the patient’s spontaneous breathing if present. This means that the ventilator tries to synchronize mandatory inflation with some, if not all, neonatal breathing efforts, thereby reducing asynchrony to some extent. However, there is still a risk of asynchrony (10–85%) due to a mismatch between respiratory rates, efforts (trigger), inspiratory time (Ti), and inadequate pressure support. This asynchrony can be somewhat improved by the AC mode (also known as synchronized intermittent positive pressure ventilation or patient-triggered ventilation), in which ventilator inflation is triggered by the spontaneous breathing effort of the patient. This mode supports all detected breaths with preset pressure (or V_T_). If there are no spontaneous breathing efforts, the ventilator will start inflation at a set backup rate. This mode avoids the issue of inspiratory asynchrony; however, due to time cycling, expiratory asynchrony may persist.

The PSV mode is similar to the AC mode, except that it is flow-cycled. It supports all patient-triggered breaths with preset pressures. The inspiration is terminated when the inspiratory flow declines to a preset threshold. Thus, it eliminates the inspiratory hold and possibly provides better synchrony. This mode requires setting a backup rate for apnea, which can be achieved by adding SIMV, or can be a feature within the PSV mode in some ventilators. Although theoretically, the assist modes (ACV and PSV) seem better than SIMV alone, there is little evidence to support this [9, 10]. A recent meta-analysis suggested that the AC mode possibly helps in slightly early weaning (mean difference: 22.7 h, 95% CI 1–44 h) compared to SIMV without a significant effect on other clinical outcomes [10].

## Volume-Targeted Ventilation

Evidence suggests that the addition of VTV to PLV significantly reduces BPD, intraventricular hemorrhage (IVH), air leaks, duration of ventilation, and composite outcomes of death and BPD [11]. Despite the strong evidence, the application of VTV in neonatal units is far from universal. This is probably due to the use of so-called universal ventilators, which may lack the accuracy required for the delivery of tiny V_T_ required for extremely low birth weight (ELBW) infants, along with the reluctance of clinicians to move away from the comfort zone of using the traditional mode (PLV) [7, 8]. Older and universal ventilators often measure V_T_ at the proximal end, thereby grossly underestimating V_T_. With current dedicated neonatal ventilators, it is possible to measure V_T_ at the patient end of the circuit, thereby making it more accurate. In VTV, the pressure changes according to the compliance of the lung to ensure the set V_T_ delivery to the airway. This means that with improved lung compliance, PIP is automatically reduced, resulting in real-time weaning. This feature makes it attractive for weaning, including the period immediately after surfactant administration. VTV has variable nomenclature according to ventilator manufacturers; therefore, users should be conversant with the ventilator used in their units. The commonly used terms for VTV are VG and pressure-regulated volume control (PRVC). These modes are essentially the same in principle, with slight variability in the way V_T_ for the next breath is calculated. In VG, the user chooses a target V_T_ based on the lung condition and the pressure limit. The ventilator then compares the exhaled V_T_ of the previous inflation and adjusts the PIP to achieve the set V_T_. An important practical consideration is that the user must attempt to reduce peri-endotracheal tube leaks. Although recent ventilators compensate for leaks, in practice, leaks of more than 30–40% make V_T_ measurements inaccurate and can be subject to excessive V_T_ delivery at inspiration. This can also lead to wide variability in breath-to-breath pressure, making it difficult to provide smooth gas flow delivery.

Because the main driving factor is V_T_, the choice of an appropriate V_T_ is critical for the successful use of VTV. Set V_T_ is affected by the infant’s size, postnatal age, and underlying lung disease. A general guide for selecting an appropriate V_T_ is provided elsewhere [7, 8]. Box 1 provides a general guide for VTV. Once the initial settings are deployed, it is essential to carefully monitor the infant and titrate them accordingly.

### Titration of Conventional Mechanical Ventilation

Ventilation is a blend of art and science. Unfortunately, titration of mechanical ventilation in neonates is still more of an art than science itself. Therefore, titration of ventilation requires an experienced clinician who can make an informed judgment based on meticulous bedside clinical assessment (rate, retraction, chest rise, oxygen saturation measured by a pulse oximeter or SpO_2_, etc.) coupled with real-time pulmonary graphics and transcutaneous CO_2_ or blood gas monitoring. Modern ventilators provide a large amount of data on delivered volumes, volume leaks, and compliance/resistance of the system, which can help in the real-time titration of ventilation. In this section, we briefly discuss pulmonary graphics.

Pulmonary graphics refer to the visual representation of the interplay of respiratory mechanics (pressure, flow, volume, and time), displayed in the form of waveforms (scalars) and loops [12–14]. These graphs provide real-time information about airflow, pressure, and volume changes in the lungs, allowing clinicians to assess lung function and optimize mechanical ventilation settings.

In neonates, common types of pulmonary graphics include pressure–volume (P–V) loops, flow–volume (F-V) loops, and time-based scalars (time against pressure, flow, and volume). Of these P–V loops, F-V loops and flow-time scalars are commonly used in clinical practice. We have provided a typical P–V loop, F-V loop, and flow-time scalar (Fig. 1A–C). The graphics can help in the early identification of air trapping (Fig. 1D-F) and hence guide clinicians in adjusting positive end-expiratory pressure (PEEP) and flow rate without waiting for chest radiographs. Similarly, peri-endotracheal tube leaks could be easily identified (Fig. 1G-I) and corrected in real-time. These graphics can help assess the response in real-time (for example, applying cricoid pressure in case of a peri-endotracheal tube leak will be reflected immediately on pulmonary graphics). Pulmonary graphics can also help optimize the PIP and hence avoid overdistension (Fig. 1J). They can also provide evidence of high airway resistance (Fig. 1K). Last but not least, they can provide guidance on the need for suction in the presence of airway secretions (Fig. 1L). Because the changes are reflected in real-time, they can be used to provide guidance for weaning (particularly in pressure-controlled ventilation). Although clinicians use pulmonary graphics, there are no clinical trials to determine whether their use actually results in better outcomes [14]. In the authors’ experience, they help clinicians make “informed decisions” in the titration of mechanical ventilation. Nevertheless, there is a need for RCTs to provide definitive scientific evidence for their utility to impact clinical outcomes.

#### High-Frequency Ventilation

HFV delivers a very small V_T_ (less than the anatomical dead space) to the lungs at extremely rapid rates (up to 900 per minute). Since volutrauma is the major factor for VILI, this mode aims to reduce VILI with less V_T_, while maintaining constant alveolar distending pressure and improving gas exchange. The three main ventilator modalities used to provide HFV are HF oscillatory ventilation (HFOV), HF jet ventilation (HFJV), and HF flow interruption (HFFI). The first two are commonly used in neonates and are discussed here.

#### High-Frequency Oscillatory Ventilation

HFOV is generated using a specialized ventilator that produces rapid, small-amplitude oscillations (up to 900 per minute) using a piston or diaphragm within the ventilator to rapidly shift air in and out of the lungs. A constant distending pressure or CDP (known as mean airway pressure or MAP) is set, which helps in alveolar recruitment and oxygenation. Amplitude and frequency are mainly responsible for ventilation, which is independent of oxygenation. V_T_ is very low (1–3 mL/kg), and the mechanism of V_T_ delivery is very different from that of conventional ventilators. There are many postulated mechanisms for the physiological basis of its function, including alveolar pendelluft, cardiogenic mixing, Taylor dispersion, and molecular diffusion [15, 16]. In clinical trials, HFOV has been used as a primary as well as rescue modality [17, 18]. When used as the primary elective mode in preterm infants, it has been shown to have a slight benefit in composite outcomes (death or BPD) and retinopathy of prematurity; however, the evidence is very uncertain [17]. Owing to this uncertainty over its benefits, the need for specialized equipment to deliver HFOV, and advancements in CMV technology and strategies, its use as the first-line mode in neonates is very limited. It is commonly used as a rescue mode in clinical practice in situations of actual air leaks or when air leaks are highly likely, for example, in hypoplastic lungs or the requirement of very high PIP in extremely premature infants. However, data to support or refute its superiority (or inferiority) over conventional ventilation are limited [18, 19].

#### High-Frequency Jet Ventilation (HFJV)

The HFJV uses short rapid (240–660 per minute) pulses of pressurized gases that are released as a jet, either directly into the endotracheal tube or into the Y-piece of the circuit. In contrast to HFOV, exhalation is passive, and to minimize the risk of gas trapping, lower operating frequencies are often applied. The settings of the conventional ventilator (PIP, PEEP, Ti, and respiratory rate or frequency or *f*), which is used in tandem with HJV, contribute to a MAP comparable to that of CMV. The inspiratory times are very short (0.02 to 0.03 s) with prolonged expiratory times, making this ventilator very effective in the management of air leaks. This mode is gaining popularity in extremely premature neonates (22–26 weeks), although there is limited data on its efficacy and safety [4, 15, 19].

### Extubation Readiness

As mentioned earlier, invasive ventilation is a major reason for VILI; therefore, ventilation strategies are directed toward gentle ventilation for as minimal duration as possible. Once an infant is intubated, a clear and written plan for extubation is required. Infants should be assessed regularly for extubation readiness. Unfortunately, there is no ready-made “one size fits all” model or single tool for assessing readiness for successful extubation. Lower gestation, sepsis, pre-extubation acidosis, pre-extubation high fraction of inspired O_2_ (FiO_2_), and higher respiratory distress severity scores were associated with an increased risk of extubation failure [20]. Many trials have used the spontaneous breathing trial (SBT) test to predict extubation success. A recent systematic review suggested that SBT in preterm infants can accurately predict extubation success, but not extubation failure. Therefore, there is a lack of evidence supporting its use as an independent predictor of extubation failure in premature infants [21]. Instead of relying on a single parameter, we suggest using a checklist to assess extubation readiness (Box 2). In general, the condition for which the infant was intubated should have been resolved, and the infant should be on minimal settings (PIP 12–15 cm H_2_O, PEEP 5–6 cm H_2_O, FiO_2_ < 0.30) and should have good spontaneous efforts. Infants < 32 weeks should preferably be extubated to nasal intermittent positive pressure ventilation (NIPPV) to improve extubation success. Postextubation care is a critical determinant of extubation success; therefore, appropriate airway management, positioning, physiotherapy, good nursing care, humidification, nasal care along with appropriate NIV support are crucial.

**Box 2:** Extubation criteria

1	Primary pathology has improved (FiO_2_: < 0.3, PIP: 14–15 cm H_2_O, PEEP: 5–6 cm H_2_O, RR < 60 bpm, no retractions, normal blood gas and electrolytes)
2	Off sedation for at least 6 h (spontaneous rates > set rates)
3	No hemodynamically significant PDA/if previously documented PDA, repeat Echo for closure
4	The chest radiograph does not show areas of atelectasis
5	Absence of nasal injury
6	Continuation of caffeine or loading dose of caffeine 24 h prior if < 32 weeks gestation
7	High risk of airway edema? If yes, at least 1 dose of dexamethasone given 4 h before extubation
8	Hematocrit > 30% and off inotropes
9	Nil per oral for at least 2 h before extubation
10	Parents and bedside nurses are informed about the extubation plan

Currently, the focus in neonatology has shifted from survival to “intact survival.” Preterm infants on invasive mechanical ventilation are at an increased risk of sepsis, ventilator-associated pneumonia, air leaks, BPD, and adverse neurodevelopmental outcomes [22]. Therefore, strategies to prevent such complications should be developed and implemented from birth. In general, invasive mechanical ventilation should be avoided as much as possible. If needed, gentle lung ventilation for as minimum as possible duration is recommended. Apart from gentle ventilation, gentle resuscitation, appropriate antibiotic use, early caffeine use, early surfactant administration (if needed) through less-invasive surfactant administration (LISA), Vitamin A supplementation, exclusive human milk feeding with increased energy uptake, avoiding excessive positive fluid balance, and maintaining oxygen saturation between 90 and 95% are evidence-based strategies for BPD prevention. A detailed description of evidence-based recommendations as per the various stages of BPD is beyond the scope of this article and is provided elsewhere [22–24].

## Conclusions

Noninvasive ventilation is the recommended first-line respiratory support in neonates with respiratory distress. However, for neonates in whom NIV fails or is less efficacious or contraindicated, the timely initiation of invasive mechanical ventilation is crucial. For neonates requiring invasive ventilation, patient-triggered synchronized modes (SIMV + PS or AC) with VTV are preferred. The use of pulmonary graphics along with meticulous bedside clinical assessment can aid clinicians in making informed decisions on the titration of mechanical ventilation. Current evidence does not support the routine use of HFV as a primary respiratory support. The IMV duration should be limited to a minimum possible extent. Timely protocolized extubation and good nursing care are the key to successful extubation. Box 3 summarizes the key messages for various modes of invasive mechanical ventilation in neonates.

**Box 3:** Key messages

For infants requiring invasive ventilation, volume-targeted ventilation (SIMV + PSV or A/C mode with Volume Guarantee) should be preferred to minimize lung injury
Pulmonary graphics may aid in making informed decisions while titrating invasive ventilation in neonates
High-frequency ventilation should be reserved as rescue support reserved for air leaks and severe lung disease
A protocolized structured weaning approach should be used to improve extubation success
Good nursing care and meticulous clinical examination are the keys to successful ventilation

## Data Availability

The data used in this review are already in the public domain.

## References

[1] De Luca D, Tingay DG, van Kaam AH, et al. Epidemiology of neonatal acute respiratory distress syndrome: Prospective, multicenter, international cohort study. Pediatr Crit Care Med. 2022;23:524–34. 35543390 10.1097/PCC.0000000000002961

[2] Chen L, Li J, Shi Y. Clinical characteristics and outcomes in neonates with perinatal acute respiratory distress syndrome in China: a national, multicentre, cross-sectional study. eClinicalMedicine. 2023;55:101739. 10.1016/j.eclinm.2022.101739. 36386029 10.1016/j.eclinm.2022.101739 PMC9661498

[3] SUPPORT Study Group of the Eunice Kennedy Shriver NICHD Neonatal Research Network, Carlo WA, Finer NN, Walsh MC, et al. Target ranges of oxygen saturation in extremely preterm infants. N Engl J Med. 2010;362:1959–69. 10.1056/NEJMoa0911781 PMC2891970 20472937

[4] Dumpa V, Avulakunta I, Bhandari V. Respiratory management in the premature neonate. Expert Rev Respir Med. 2023;17:155–70. 36803028 10.1080/17476348.2023.2183843

[5] Bhandari V, Black R, Gandhi B, et al. RDS-NExT workshop: consensus statements for the use of surfactant in preterm neonates with RDS. J Perinatol. 2023;43:982–90. 37188774 10.1038/s41372-023-01690-9 PMC10400415

[6] Rocha G, Soares P, Gonçalves A, et al. Respiratory care for the ventilated neonate. Can Respir J. 2018;2018:7472964. 30186538 10.1155/2018/7472964 PMC6110042

[7] Martin Keszler M, Abubakar K. Volume-targeted ventilation. Semin Perinatol. 2024;48(2):151886. 10.1016/j.semperi.2024.151886. 38553330 10.1016/j.semperi.2024.151886

[8] Keszler M. Volume-targeted ventilation: one size does not fit all. Evidence-based recommendations for successful use. Archiv Dis Childhood–Fetal Neonatal Ed. 2019;104(1):F108–12. 10.1136/archdischild-2017-314734. 10.1136/archdischild-2017-314734 30068668

[9] Greenough A, Rossor TE, Sundaresan A, Murthy V, Milner AD. Synchronized mechanical ventilation for respiratory support in newborn infants. Cochrane Database Syst Rev. 2016;9:CD000456. 27581993 10.1002/14651858.CD000456.pub5 PMC6457687

[10] Batra D, Jaysainghe D, Batra N. Supporting all breaths versus supporting some breaths during synchronised mechanical ventilation in neonates: a systematic review and meta-analysis. Arch Dis Child Fetal Neonatal Ed. 2023;108:408–15. 36631252 10.1136/archdischild-2022-324464

[11] Klingenberg C, Wheeler KI, McCallion N, Morley CJ, Davis PG. Volume-targeted versus pressure-limited ventilation in neonates. Cochrane Database Syst Rev. 2017;10:CD003666. 29039883 10.1002/14651858.CD003666.pub4 PMC6485452

[12] Lal MK. Neonatal Pulmonary Graphics: A Clinical Pocket Atlas. Semin Fetal Neonatal Med. 2015;20:279.

[13] Rocha GM, Soares PO. Interpreting real-time pulmonary graphics in neonatal invasive conventional mechanical ventilation. Turk Arch Pediatr. 2021;56(4):285–94. 10.5152/TurkArchPediatr.2021.21103. 35005721 10.5152/TurkArchPediatr.2021.21103 PMC8655954

[14] Bhutani VK. Clinical applications of pulmonary function and graphics. Semin Neonatol. 2002;7:391–9. 12464501 10.1053/siny.2002.0133

[15] Ackermann BW, Klotz D, Hentschel R, Thome UH, van Kaam AH. High-frequency ventilation in preterm infants and neonates. Pediatr Res. 2023;93:1810–8. 35136198 10.1038/s41390-021-01639-8 PMC10313521

[16] Courtney SE, van Kaam AH, Pillow JJ. Neonatal high frequency ventilation: current trends and future directions. Semin Perinatol. 2024;48: 151887. 38556386 10.1016/j.semperi.2024.151887

[17] Cools F, Offringa M, Askie LM. Elective high frequency oscillatory ventilation versus conventional ventilation for acute pulmonary dysfunction in preterm infants. Cochrane Database Syst Rev. 2015;2015:000104. 10.1002/14651858.CD000104.pub4 PMC10711725 25785789

[18] Bhuta T, Henderson-Smart DJ. Rescue high frequency oscillatory ventilation versus conventional ventilation for pulmonary dysfunction in preterm infants. Cochrane Database Syst Rev. 2000;1998:000438. 10.1002/14651858.CD000438 PMC7032669 10796364

[19] Rojas-Reyes MX, Orrego-Rojas PA. Rescue high-frequency jet ventilation versus conventional ventilation for severe pulmonary dysfunction in preterm infants. Cochrane Database Syst Rev. 2015;2015:CD000437. 26474355 10.1002/14651858.CD000437.pub3 PMC7032889

[20] Fu M, Hu Z, Yu G, et al. Predictors of extubation failure in newborns: a systematic review and meta-analysis. Ital J Pediatr. 2023;49:133. 37784184 10.1186/s13052-023-01538-0 PMC10546653

[21] Teixeira RF, Carvalho ACA, de Araujo RD, Veloso FCS, Kassar SB, Medeiros AMC. Spontaneous breathing trials in preterm infants: Systematic review and meta-analysis. Respir Care. 2021;66:129–37. 32843509 10.4187/respcare.07928

[22] Gilfillan M, Bhandari A, Bhandari V. Diagnosis and management of bronchopulmonary dysplasia. BMJ. 2021;375: n1974. 34670756 10.1136/bmj.n1974

[23] Bhandari A, Alexiou S. Outpatient management of established bronchopulmonary dysplasia: an update. Semin Perinatol. 2023;47: 151820. 37777461 10.1016/j.semperi.2023.151820

[24] Dumpa V, Bhandari V. Surfactant, steroids and non-invasive ventilation in the prevention of BPD. Semin Perinatol. 2018;42:444–52. 30343941 10.1053/j.semperi.2018.09.006

